# Commenting on Aristotle with a Knife: The Heretical Anatomies of Bassanio Landi

**DOI:** 10.1007/s00048-022-00354-7

**Published:** 2023-02-23

**Authors:** Fabrizio Bigotti

**Affiliations:** 1 JMU Würzburg, Würzburg, Germany; 2 University of Exeter, Exeter, UK; 3 CSMBR, Pisa, Italy

**Keywords:** Bassanio Landi, Anatomy, Aristotle’s *De Anima*, Heretical Thinking, Sixteenth-Century Padua, Bassanio Landi, Anatomie, Aristoteles’ *De Anima*, Ketzerisches Denken, Padua im sechzehnten Jahrhundert

## Abstract

Drawing on a variety of sources, including manuscript notes and a wide variety of published material, this article offers the first analysis in English of Bassanio Landi’s works in their medical and philosophical context. I argue that while Landi’s output is characteristic of its sixteenth-century Paduan milieu, his approach to methodological questions in anatomy and the arts, as well as his paraphrase of Aristotle’s *De anima*, make it possible to locate him within the heretical tradition that stretches from Pietro Pomponazzi (1462–1525) to Paolo Sarpi (1552–1623).

## Abstract (de)

Auf der Grundlage einer Vielzahl von Quellen, darunter Manuskriptnotizen und eine breite Auswahl an veröffentlichtem Material, bietet dieser Artikel zum ersten Mal in englischer Sprache eine Analyse von Landis Werken vor ihrem medizinischen und philosophischen Hintergrund. Ich behaupte, dass Landis Werk zwar charakteristisch für das paduanische Milieu des 16. Jahrhunderts ist, dass aber seine Herangehensweise an methodische Fragen der Anatomie und der Kunst sowie seine Paraphrase von Aristoteles’ *De anima* es ermöglichen, ihn in der Mitte der häretischen Tradition zu verorten, die von Pietro Pomponazzi (1462–1525) bis Paolo Sarpi (1552–1623) reicht.

Teaching and practicing medicine in sixteenth-century Padua could end up being a rather risky business. When not dealing with undisciplined students making noise and interrupting the lectures, or demanding extra time for private dissections, professors often had to contend with academic rivalries and religious censorship. Although it was likely less intense under the Venetian authorities than elsewhere in Europe, the latter threat had existed in Padua since Pietro D’Abano (1257–1316) was tried for heresy, intensifying after the publication of Pietro Pomponazzi’s *Tractatus de immortalitate animae* (1516) (see Bylebyl 1979; Prosperi 1981; Biondi 1981; Pomponazzi 1999; Baldini 2001). Pomponazzi (1462–1524) had divorced the Aristotelian theory of the soul from the Christian dogma of the afterlife, a thesis for which he faced death threats and the public burning in Venice of copies of his book (see Craig 2014; Sgarbi 2010; Bakker & Thijssen 2007; Maclean 2005; Perrone Compagni in Pomponazzi 1999; Ferretto 2012: 21, 44–45). Not unlike Pomponazzi, who had studied both medicine and philosophy, most physicians were often at odds with demands to comply with dogma, as their very task was to understand the effects of nature on the body of their patients. In other words, they relied on evidence and verifiable experience, none of which seemed to support the separate existence of an immortal soul in the body.

## Padua 1561: A Serious Accident

Particularly bold or controversial statements on the nature of the soul and other matters on the threshold between medicine and theology could give rise to intellectual rows that occasionally turned into physical threats, as in the case of the physician Bassiano Landi:

“At the time when the most excellent physician Bassiano Landi from Piacenza was teaching theoretical medicine at the Gymnasium in Padua, many envied him and, for this reason, certain scoundrels attempted to take his life in the year 1561. These scoundrels filled a small box with gun powder and secretly put it under the chair where he used to teach publicly every day. They attached a long fuse to the small box that led out of the auditorium through some invisible fissure in the wall. They intended to light the fuse outside the auditorium so that, creeping forth slowly, the fire would eventually reach the powder contained in the small box and Bassiano would have been killed in the midst of his students and at great risk to his colleague Giunio Paolo Crasso, who was lecturing at the same time in the room directly above that of Bassiano. They would have surely succeeded in their mission if, in taking to his chair, Bassiano—who was a man provided not just with refined nostrils but with an equally excellent sense of smell—had not smelled smoke. At first, he thought it was coming from a nearby building, but, since the smell persisted, he asked his servant to inquire into the matter. Thus, the lit fuse was found, and, by following its path, they saw the small box underneath the chair that would have put an end to Bassiano’s life within ten minutes. Bassiano then moved to Venice to inquire into the plot. The Senate ordered him to take the matter with good spirits and urged him to return to his professorial duties. A reward of 2000 pounds was decreed, and authority was given to recall people from exile, and yet, despite the large sum allocated, it was not possible to discover the instigator of the plot.”^1^

Almost everything we know about the enigmatic figure of physician and philosopher Bassiano Landi (c. 1510–1562) rests on this rather picturesque episode. While a variety of discordant accounts of the attack exist, almost all the sources seem to agree that the motive was academic. According to the historian Gilbert Cousin (1506–1572), Landi had been subject to grave warnings a few days earlier. While in the company of a number of Venetian patricians, he was assaulted in the street by individuals who charged at him with an intense series of stiletto strokes. Unable to protect their master, his students managed to push him inside the shop of a nearby bookseller (Cousin 1567: 612). Although the perpetrators could not be indentified, rumours soon circulated that they were scholastic philosophers (*scholasticos*) who opposed Landi’s philosophical teachings.^2^ Landi had promised his readers a work “On Scholastic Controversies” (*De scholasticis controversiis*),^3^ which was to contain a detailed examination of the most important and controversial philosophical issues of the time; a manuscript treatise of his “On Methods and Orders” (*Trattato de i metodi et de gli ordini*) was also circulating within the nobility in Venice, which promised to challenge the primacy of syllogism as a method of demonstration. While both these works remained unpublished and are now lost, the fact that Landi could propose a discussion on such matters outside the official setting of the Paduan academia was sufficient to cause outrage. Tensions with colleagues were further inflamed by Landi’s demands to maintain his salary as an anatomy reader at the same level as that of Gabriele Falloppia (1523–1562) and by his reaction following Falloppia’s death in 1562, at the news of which he was said to have rejoiced. As demonstrated by Michael Stolberg, contemporary accounts refer to this incident as the alleged explanation as to why Falloppia’s pupils chased after Landi to stab him to death (Stolberg 2022: 152–163).^4^

While a valuable source, the latter explanation of Landi’s death carries less weight than the former. Indeed, threats to Landi’s life had already occurred before Falloppia’s death, not as a consequence of it. Furthermore, by 1560 Landi no longer had any serious interest in anatomy. As we shall see, his efforts were predominantly aimed at obtaining a chair in natural philosophy and theoretical medicine, acknowledging that he had nothing new to contribute to anatomy, a field in which he could hardly pose a serious threat to masters such as Falloppia. In short, if we wish to get a glimpse into the reasons that led to his assassination we ought to look closely at Landi’s works and their philosophical content.

The most contentious works were allegedly a series of private lectures he delivered on Aristotle’s natural writings, as these sparked the interest of the scions of the Venetian nobility who now had access to heterodox readings of the official curriculum (Ferretto 2015: 51–60). Landi concentrated in particular on Aristotle’s *De anima* and, in line with Pomponazzi’s reading thereof, denied the possibility of using Aristotle’s authority to underpin the Christian dogma of the immortality of the soul. According to Antonio Polo, a contemporary source, it was Landi’s deeply heretical stance that prompted the series of violent acts that eventually occurred.^5^ Indeed, Landi was assaulted by masked killers in Padua on the night of 21 October 1562 and, wounded by seven stiletto strikes to the head, succumbed to his injuries ten days later (Ongaro 1998: 33; Ferretto 2012: 19–20).

Drawing on a variety of sources, including manuscript notes and a broad sample of published material, this article offers the first analysis in English of Landi’s works in their medical and philosophical context. Previous scholarship—almost exclusively represented by Ferretto’s short monograph in Italian—has focused on a contextual analysis of Landi’s work while what I offer here is a comprehensive look at the entirety of his output by providing new material to frame the development of post-Vesalian anatomy and the evolution of Venetian Aristotelianism in particular. In this regard, I argue that while Landi’s output is characteristic of its sixteenth-century Paduan milieu, his approach to questions of method and his commentary on Aristotle’s *De anima* make it possible to locate him within a heretical tradition that stretches from Pietro Pomponazzi (1462–1525) to Paolo Sarpi (1552–1623).

## An Outline of Landi’s Life and Works

Little is known about Landi’s life. The sources and testimonies that remain have been painstakingly gathered by the Italian scholar Silvia Ferretto (2012). Landi was born in Piacenza—in the titles of his few works he calls himself as *Placentinus* and his two wills confirm that he had several possessions in the region. Contemporary accounts agree that Landi was a very talented classicist. He had taught Latin and Greek in Reggio Emilia from 1535 to 1537, from which we can gather that he was probably born around the first decade of the sixteenth century (Ferretto 2012: 25–37). He completed his medical studies in Padua, under Giovanni Battista da Monte (1498–1551), and graduated on 31 August 1542 under Vittore Trincavelli (1496–1588) (Ongaro 1998: 34).

An expertise in ancient languages is reflected in the majority of Landi’s works, even the most strictly anatomical ones, such as his first publication: *De humana historia libri duo* (Basel 1542). In it, the order of bones and organs in the body is presented discursively, in a direct address to the reader, rather than in the usual dry and scholastic style typical of medical commentaries of the time. In the preface, Landi declares the need to approach anatomy in this way because available translations of Greek words into Latin are often weak and misleading (*molliter translatas esse, ac parce detortas*), while also highlighting the novelty of the project (Landi 1542: 3 [unnumbered]). The book is written in a style never previously attempted by Latin writers. Its originality stems from the fact that, unlike other commentaries, Landi had to come up with a new terminology, which he expected to receive criticism (*reprehensione aliqua vacare vix posse*) (Landi 1542: 3). While Landi’s approach was not uncommon for early sixteenth-century humanist physicians, committed to gaining access to the texts of the ancients through the most advanced philological instruments, by midcentury it had become progressively less popular. Following Andreas Vesalius’ (1514–1564) critiques of Galen and authority more widely, the philological “anatomy of the text” had lost ground against the more spectacular anatomy of the body (see Landers 2012; Kusukawa 2012). As opposed to this later approach, Landi’s preference for the anatomy of the text is visible everywhere, especially in his extensive use of Greek words to retrace the origins of Latin anatomical terminology. In conjunction with the first step in his Paduan career as a professor of practical medicine *in secundo loco* (1543), Landi published another medical work, the *Iatrologia* (1543), wherein a method to “discover” (*methodus inveniendi*) diseases is presented to the reader in the form of a dialogue between two of Landi’s friends, Jacopo Bonfadio and Pietro Cassio (see Ferretto 2012: 37–47).

The *Iatrologia* proved to be another controversial work for the Paduan physician. Dialogues on method were uncommon at the time. Those who had used this unusual literary form did so either because they operated outside established academic networks (like Girolamo Fracastoro, 1478–1553) or because their works were published posthumously from a collection of student notes (as in the case of Giovanni Battista da Monte, 1498–1551) (Ongaro 1965: 265–266; Ferretto 2012: 25–37). As a benchmark of scholastic philosophy, methods were supposed to follow a strictly deductive order of exposition. On the contrary, approaching method in a literary form was a trait shared by Platonic philosophers and became widespread amongst the followers of Petrus Ramus (1515–1572), the French humanist who sought to replace Aristotle’s logic with a new method for teaching the arts (see Knight and Wilson 2019; Ingemarsdotter 2011; Freedman 2000; Oldrini 1997). Ramus and his followers advocated for a new foundation for method, no longer based on syllogism, but on dialectics—that is a form of knowledge achieved by adopting the rhetorical means of dividing a definition into many sub-definitions and then reorganising them into an articulated and consistent discourse which allowed humanists to present their students with a method to connect the arts and sciences. Landi’s claim that the originality of his works lay “in the style, clarity, and order of exposition rather than in the doctrine itself,”^6^ belongs to this very climate and thus betrays a more oblique intent. As an example, in the *Iatrologia*, Landi praises his teacher Giovanni Battista da Monte for passing on to him “without fault and errors […] the proper way of medicine, which has remained unknown thus far.”^7^

This “proper way” becomes clear in the dialogues of the *Iatrologia*, as Landi proposes that the students should not only recognise but “discover” diseases (*quendam morbos inveniendi methodum continet*) by using a method called *divisio *or *distributio rei in proprias differentias*—that is the rhetorical techniques of *inventio* and *dispositio* discussed above that were familiar to humanists via the works of Cicero and Quintilian.^8^ While the acknowledgement of such techniques as a proper methodology for teaching medicine was already a contentious and heterodox position, Landi seems to have applied it both in his anatomies and his philosophical lectures, as discussed below. Concurrent with his promotion to the second chair of natural philosophy (1545) followed by the first chair in theoretical medicine two years later (1547), replacing Antonio Fracanziano, Landi’s heterodox positions became less extreme. These professional advancements coincided with an intense period of philosophical teaching, culminating in the publication of the *Opuscula* (Padua, 1552), a collection of philosophical digressions (*ekphraseis*) on the nature of motion, time, and place with a booklet on bloodletting (*de vacuatione*) and the promise to soon publish a large commentary on Hippocrates’ *Aphorisms*, of which only the preface is offered in the *Opuscula*. These publications were followed four years later by another booklet on *De incremento* (Venice, 1556), which is reminiscent, in form and content, of Pomponazzi’s work *De nutritione et auctione*, published in Bologna in 1521.

According to Theodor Zwinger (1533–1588), a Swiss naturalist and physician who had been trained in Ramus’ anti-Aristotelian methods, Landi’s philosophical publications and mentoring were instrumental in promoting a new approach to Aristotle’s texts (Zwinger 1566: 23–24; Ferretto 2012: 15–18). Landi presented them in a simple, clear, and less dogmatic style, which made Aristotle’s philosophy accessible to the young and those not usually attracted to his writings. Zwinger’s account offers a glimpse into Landi’s larger philosophical undertaking, which was aimed at establishing himself as a theoretical physician and eventually led him to the major task of commenting on Aristotle’s *De anima*—the cause, according to contemporary sources, of his death. Unusual both in bulk and approach, Landi’s commentary is in fact a philological translation with short paraphrases of difficult passages: nothing could be more different from the lengthy and bulky translations and commentaries of Aristotle’s works that were standard at the time. The *De anima* paraphrase was published posthumously, seven years after Landi’s death (1569), and marks the crowning moment of the sixteenth-century physician and anatomist’s career, whose output remained controversial from beginning to end.

## Outlines of Landi’s Approach to Method

To unpack some of the motivations behind Landi’s provocative approach, it is necessary to offer an analysis of the philosophical background of his works, especially the *Iatrologia *and the* Opuscula* as illustrated by three private letters^9^ addressed to him by the nobleman Sebastiano Erizzo (1525–1585), one of Landi’s pupils in Padua during the period 1543–1545. Erizzo’s letters address certain critiques that Landi had made of his pupil’s manuscript, *Dell’instrumento et via inventrice degli antichi* (“The Instrument and Method of Invention of the Ancients”), eventually published by Erizzo in 1554. Both in his letters and works, Erizzo offers a glimpse into Landi’s approach to method and the influence it had on his pupils. In the first letter (15 November 1551), Erizzo addresses Landi’s criticism that the title is unclear and should be revised to read *Della prestantia dell’istrumento divisivo *or *Dell’eccellenza del metodo divisivo* (“On the Efficacy/Excellence of the Divisive Method”). Erizzo praises his master for having introduced him to the importance of division as a method in the arts. Landi had discussed the importance of division as an instrument of discovery in his *Iatrologia*, while the *Opuscula* had demonstrated how this method could be used to present complex issues such as the nature of motion and time.^10^ As we shall see, however, Landi’s use of division was conceived to be used in conjunction with syllogism, not as a substitute for it, which explains why he criticised his pupil for the bold title of his forthcoming book. To defend himself against Landi’s criticism, Erizzo quotes passages from Plato, Aristotle, and Proclus, wherein division is praised for its capacity to lead to the discovery of new truths. In the second letter to Landi (dated 4 March 1552), Erizzo refers to Landi’s manuscript treatise “On Methods and Orders” which discussed the efficacy of division in detail and which Erizzo had consulted. Erizzo praises Landi’s hesitancy in publishing it, because the work would have been met with opposition from poorly-qualified “envious people.”^11^ Whether or not such a manuscript was in fact part of the promised work on scholastic controversies remains unclear. Landi’s propensity to use division as a method and the extent of his influence on Erizzo’s work is visible in the fact that Erizzo dedicated *Dell’instrumento* to Landi who, in Erizzo’s words, had first introduced the possibility of thinking of “division” and “order” as methods of equal value to syllogism and apodictic demonstration.

## Division as a Method in Context

To fully appreciate Erizzo’s praise, it is necessary to explore the meaning and applications of this method within the context of the Renaissance debate over the best method with which to teach the arts and sciences (Bigotti 2021; MacLean 2002). The debate featured prominently in the dispute between physicians (*medici*) and philosophers (*philosophi*) over the value of logic and demonstrative methods in the arts. In medicine, the debate hinged on Galen’s definition of medicine as an art and the possibility, discussed in the *Ars medica*, of using division (*divisiva methodus*) and the definitive method (*methodus definitiva*) in addition to the more usual induction (*compositiva methodus*) and deduction (*resolutiva methodus*) (Bigotti 2019: 40–45; Ferretto 2012: 99–118). Galen’s claim with regards to the *divisiva methodus* was supported and defended by many physicians, including Landi’s master, Giovanni Battista da Monte, as a means of providing a clearer exposition of medical knowledge and—more importantly—to guide practice to better results. In essence, division was a method of exclusion (*either*/*or*) in which the most universal properties were subdivided into pairs of sub-definitions following the inclusion of species into genera until the characteristics that most clearly approximated the essence of the object to be defined were reached. As an example, in defining iron, one could start by enumerating the most universal properties of a being and then, upon deciding which one of the opposite pairs better suits the object, reach the object’s intended definition (for instance: anything that is, is *either* substance *or* accident; if it is substance, is *either* corporeal *or* incorporeal; if it is corporeal, is *either* eternal *or* corruptible; if it is corruptible, is *either* a living being *or* not a living being, if it is not a living being it is a mineral. If it is a mineral, it is *either* a, b, *or* c, and so forth). In pursuing such a method, physicians thought they could reach a definition of the single case and thus direct the therapeutic action to a specific goal.

More generally, the debate on method affected the use of authority with the possibility, defended in particular by the humanists, of developing and adding new methods to the traditional curriculum, which was seen as a direct challenge to the scholastic vision of science. To humanists like Niccolò Leoniceno (1428–1524) and Janus Cornarius (1500–1558), the discussion on the best method to teach the arts was also a proxy for dethroning scholasticism with the aid of the new instruments provided by philology, thus bypassing the lengthy questions of scholastic philosophers by drawing directly from the original texts of Hippocrates, Plato, Aristotle, and Galen (Mammola 2012: 69–76, 107–114; Garin 1994; Siraisi 1987). In this sense, the revival of “the ancients” went along with an attempt at subverting Aristotle and his methods as enshrined in academia by three centuries of scholastic philosophy. By the same token, this penchant for philology was viewed with suspicion by church authorities in that it seemed to mirror the demands of reformist theologians who, in their attempt to reform Catholic doctrines, encouraged a more direct access to the sacred texts, often grounding their demands on the philological need to better grasp the meaning of the scriptures. On a practical level, the humanists’ argued that traditional methods were ineffective in medicine, as they were of no help in curing diseases. This meant that medicine could not be considered a science granted *a priori* via demonstrative principles, but had to acknowledge its contingency and failures and seek alternative paths to deal with its objects: healthy and diseased individuals. In so doing, the humanists advocated the introduction of new methods that, initially seen as ways of teaching, were eventually advocated as a full-blown alternative to the established Aristotelian demonstration. As previously mentioned, this was one of the main reasons behind the diffusion of Ramist methods in sixteenth-century Europe, whose acceptance was particularly widespread amongst reformed thinkers. In both the humanist and Ramist approaches, the traditional relationship between medicine and philosophy, seen as a subordination (*subalternatio*) of the former to the latter, was turned upside down, with the practical arts now reclaiming their autonomy from philosophy and theology. Ultimately, the goal was to avoid the question of subordination altogether or at least redefine the relationship between practical and theoretical disciplines (Mammola 2012: 134–152; Ferretto 2012: 99–118). Be this as it may, by the mid of the sixteenth century, the scholastics were regaining ground, and eventually the possibility of upholding different methods was dismissed by Jacopo Zabarella (1533–1589) and the late-century anatomists, who reinstated the *subalternatio* of practical to theoretical disciplines (Mammola 2012: 216–240; Mikkeli 1992: 30–40).

It is within this context that Landi’s approach to method with regards to his teacher Da Monte and his pupil Erizzo must be located. While Da Monte had advocated for the divisive method as a means of better defining the range of applicability of abstract medical notions to everyday practice, Landi argued for it as an instrument of “invention,” namely a method for the discovery of diseases. Landi’s *Iatrologia* sets out to explore the limits and possibilities of such a method, which is no longer secondary to syllogism but works in conjunction with it, as a legitimate and alternative method to be used in medicine. Erizzo seems to have taken the task a step further, by expanding on Landi’s claim and engaging in a fuller account as to how to adopt the divisive method as a proper demonstration, while refuting the passages where Aristotle contended that division presupposes the object to be defined and is therefore weak and incorrect (Vanhaelen 2016).^12^ Philosophically, for Erizzo, it was also a matter of defending Plato and the Neoplatonic tradition against the vitriolic criticism of the scholastics. In short, Landi made the first step in enfranchising the divisive method as an instrument of invention, while his pupil expanded upon this claim by showing the full implication of Landi’s approach.

## Applications of Division: Landi’s Anatomical Lectures

**Fig. 1:**
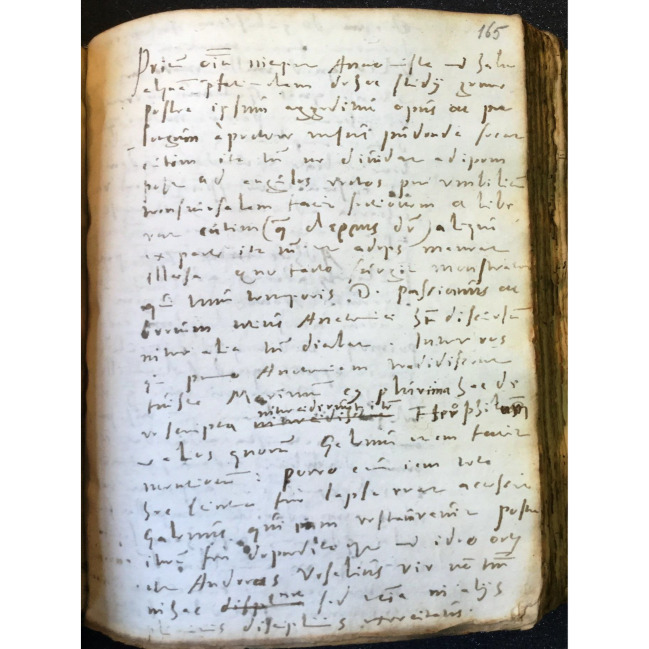
First page of Bassiano Landi’s manuscript anatomical lectures as collected by an anonymous German scholar in Padua (c. 1560). (Erlangen Nuremberg Library, Ms 909, c. 165r n.d.)

A rare opportunity to look at the application of Landi’s concept of method is provided by Michael Stolberg’s discovery of a set of manuscript anatomical notes collected by a German student of Landi (2018). The manuscript (Ms 909, cc. 160r–205r, Fig. 1), kept at the University Library of Erlangen-Nuremberg in Germany, dates to around 1560, based on the fact that, at various points, Landi reports on the criticisms directed at Vesalius by his colleague and rival Matteo Realdo Colombo (*Renaldus* in the text), whose *De re anatomica* was published in 1559.^13^ Most likely the anatomical dissections took place privately, because, despite being a pupil of Vesalius, Landi never held the chair of anatomy in Padua. Of note is also Landi’s role in it, which was that of a “demonstrator” (*ostensor, demonstrans*) assisted by an anatomist (*sector, anatomista*), who is identified as Paolo da Urbino.^14^ This setting, in which *ostensor* and *sector* entailed different roles, meant that Landi was in charge of presenting his students with a detailed oral account of the parts of the human body which were then dissected and illustrated in detail by the anatomist. This (largely pre-Vesalian) way of going about anatomy best suited Landi’s abilities and method of teaching, an aspect confirmed by the fact that he chose to proceed to account for the number and names of the parts of the body not in the order the parts appear to the viewer, but by applying the method of division illustrated above; in short, by following a logical rather than a topological order.

Landi begins his lecture with a brief introduction to the history of anatomy, highlighting the roles of Galen and Vesalius in reviving the discipline after it had fallen into oblivion, and then goes on to point out the various organs in the abdomen by declaring their relation to each other and their position in an order of genus-species.^15^ The Latin verb *dividitur* is used in the text to highlight the logical procedure followed by Landi in enumerating the three regions of the epigastrium (upper, median, lower), the difference between simple parts (*partes simplices seu similares*) as well as in declaring the various types of fat (*adeps*) surrounding the part. The division of genera into species is meant to present the students with a method to memorise the number and connections of anatomical parts before they are seen and recognised as such on the anatomy table. To clarify this mental path, so that it matches with the readings of Galen and the ancients, Landi often clarifies the Latin anatomical lexicon with the Greek word, and provides a translation for it, whenever necessary criticising other, incorrect, translations.

In many ways, Landi’s divisions are akin to the method used by anatomists such as Loys Vassé (died 1550) whose texts do not provide illustrations but diagrams and tables that list and classify the various organs of the body so as to aid in memorisation.^16^ This process is helped along by the inclusion of the etymology of the names for each structure, and Landi dwells in particular on the etymology of muscles and the aorta.^17^ Judging from its use in an anatomical setting, it seems that the divisive method was mostly used to present students with previously known concepts, rather than for the discovery of new ones. Even in this form, Landi’s divisions were not always accurate, and the anonymous German student complains about Landi being negligent (*aliqua negligentia sic locutus*) in the proper naming of the peritoneum fibres, which he kept calling “straight” (*fibrae rectae*) when they were clearly transversal (*tranversae*).^18^ Moreover, the overall presentation must have seemed rather wordy, as the student protests that some dissections were too short for him to appreciate them fully, especially in the case of the uterus and the muscles of the peritoneum.^19^ At times, Landi invites his students to form a mental image of the organs to be inspected by deducing their shape from the structure of the surrounding organs, as in the case of the bladder,^20^ whereas, at other times, he engages in experimentation, for instance when the anatomist shows how the ureters graft into the bladder by inserting a silver needle in them. On the whole, Landi’s anatomy was rich and articulate, covering the organs of the abdomen, the uterus, the bladder, the stomach, the liver, the muscles of the thorax, the heart and its veins and arteries, the lungs and the larynx, which were shown comparatively in the body of an old woman and several dogs, using a human skeleton for the localisation of the structures when these were not directly accessible to inspection.^21^ The most interesting parts of the manuscript are probably when Landi reports Vesalius’ opinion on contemporary anatomical controversies, quoting from his teacher’s works, such as Vesalius’ *Epistula* and *Epitome*, as well as from his predecessor Antonio Fracanziano.^22^

The function and purpose of each anatomical structure are touched upon only incidentally, but a clear reference to Aristotle’s *De anima* is made when introducing the structure of the uterus and discussing male and female as the principles of generation.^23^ Indeed, as Landi noted in *De humana historia*, anatomy is an integral part of natural philosophy, Aristotelians having made the most accurate contributions in the field (Landi 1542: 3).^24^ A commentary on Aristotle’s philosophy was therefore the most suitable place to discuss controversial topics, such as the seat of the rational soul and the links between cognition, sense perception, and life processes, which he could address only tangentially in the context of an anatomical lecture. Be this as it may, Landi makes it clear that, by 1542, he had already prepared a commentary on Aristotle’s *De anima* (Ibid.,: 5),^25^ as he not only makes reference to it in *De humana historia*, but also already makes certain very bold claims with regards to the soul in it—for instance arguing that the brain was not only (as was commonly believed) the seat of the cognitive faculties (*phantasia* and *sensus communis*), but of the entire mind (*mens*), an assertion further strengthened by locating various faculties of the soul (memory, intellect, imagination) in the different ventricles of the brain. As Landi notes, this is reflected in the fact that, in the course of a disease, one faculty can be impaired without the other being affected (Ibid., 57–58).

Such claims *de facto* entailed that the soul was a material substance that could be spatially located in the body, and that it was thus mortal. In a wider sense, both conclusions can be regarded as an unwarranted application of the divisive method which requires to articulate as different objects concepts and terms not sharing in any common definition—as in the case of the brain, whose faculties of memory, intellect, and imagination, not sharing in any common definition, ought to be located in different anatomical seats (Ibid., 61). As we shall see below, in applying division as a method of analysis and anatomical inspection, Landi drew inspiration from Aristotle, who (he believed) had himself made use of such a method in his definition of the soul (Landi 1569: 15–16). And yet, while Landi’s already heterodox opinions engendered by the use of division could easily pass unnoticed as part of standard anatomical teaching, they were destined to arouse quite a different reaction when converted into a full-blown attempt at challenging the *status quo* of the relationship between body and soul as codified in scholastic teachings. As anticipated, such relations were complicated further by the fact that the Aristotelian framework used to explain them was perceived as increasingly incompatible with the use that church authorities made of Aristotle.

## Aristotle’s *De Anima*: Readership, Controversies, and Heresies

Indeed, a Christian reader of Aristotle’s *De anima* could find within it plenty of inspiration for heretical thoughts. The work deals with the characteristics of life and its associated processes, understood as the development of a principle (ψυχὴ, *anima*) which, articulating itself through different material components, shapes each of them as an instrument (ὄργανον) fit for the soul’s conservation. The instruments thus shaped (or, better, *informed*) relate to each other in a hierarchical order that reflects the subordination of the various functions in a system. The result is a unitary structure that is self-sufficient and—as it is instrumental to the implementation of the system—is called “organic body” or simply organism. As the ability to shape such a system (for instance in the embryo), the principle exists “potentially” (*esse potentiale*); its “actuality” (*esse actuale*) being nothing but the fully functional system. Thus, insofar as actuality predates potentiality, the soul is the system itself, as the formal activity or programme by virtue of which the organic unity of a body susceptible to being so organised is realised.^26^

Understood as a system, the soul is a formal principle independent from matter; however, the very fact that it depends on the instrumental activity of bodily parts for its existence makes the soul bound to the destiny of the body, and therefore corruptible and mortal. This extends to the cognitive process as well, including the self-awareness that—according to Aristotle—takes place by reflecting upon images (φαντάσματα) that stand for, and at the same time replace, external sensory stimuli. These images can either be a false or true representation of reality, but it is predominantly by relying on them that individuals base their knowledge of the external world.^27^ However, since among all the notions that humans are capable of grasping some are contingent while others are universal and necessary, these latter notions must predate and pre-exist the individual perception thereof. They reflect the ultimate metaphysical structure of reality and, as such, are not subject to the mediation of the senses and are thus intuitively known as either existing or not existing.^28^ To put it simply, the metaphysical constituents of reality are “simple notions” (τὰ ἀσύνθετα, τὰ ἁπλᾶ) and are thus independent of time and space, while the notions we gain from everyday experience are “composite notions” (σύνθετα) in that they refer to the now and here of contingent existence and are therefore prone to error.^29^ How in effect such notions exist *per se* outside the historical community of individuals who live and talk about them has always represented one of the most difficult chapters in Aristotle’s *De anima* and probably the most debated topic in the history of Western philosophy. Aristotle defines the capacity to grasp such notions as something “divine” (θέιος),^30^ and following in his footsteps some interpreters, such as Alexander of Aphrodisias (died 200 AD), identified it with God, effectively divorcing it from the individuality of the human being (Kessler 2011). Be this as it may, and considering the question as a whole, Aristotle’s view seems irreconcilable with the possibility of an individual afterlife.

Historically, however, the debate had reverberations that went beyond the correct interpretation to be given to his philosophy. In fact, to the extent to which Aristotle’s metaphysics were embedded in Christian theology, the two were considered as different aspects of the same problem, which explains why certain interpretations of Aristotle could be allowed, while others—which conflicted with Christian dogma—were censored or discussed as a mere hypothesis. It is worth bearing in mind that the dogma of the immortality of the soul is by no means a minor one in Christian theology, whether Catholic or Reformed; it is the pivot around which the entire teachings of the Gospels revolve: “those who are last ‘in this life’ will be the first ‘in the other one’” (Matthew, 20:16) because of Christ’s resurrection. Divorcing this eschatological design from the metaphysical structure that underpinned it could cause the implosion of the entire belief system, which explains why church authorities were, quite understandably, preoccupied with preventing any attempt in this direction. Such attempts, however, intensified at the end of the fifteenth century as two important disciplines—philology and anatomy—came into their own. Both had the potential to disrupt traditional assumptions by historicising the text and the body. Philology could prove that the meaning of words is not universal but changes over time and, therefore, the presence of the same word in a different context is no guarantee of the unity of meaning in both. To reach out to the original meaning one had to put contemporary assumptions in brackets and try to work out the meaning of the text without immediately accepting the tradition accompanying that text and thus the dogmas around its interpretation. In different but complementary ways, anatomy reached the same goal, by cutting the body into parts and asking the theory to respond to the material structure of the inspected parts, rather than to the precepts of any authoritative source.

Both approaches came into full bloom by the early sixteenth century. In Padua, a stronghold of Aristotelianism but also the birthplace of modern anatomy and the scientific method, they had a particular large impact. It was in Padua that Pomponazzi started challenging not only the official interpretation of Aristotle’s *De anima* offered by the church, but opened up the possibility of doing so through the use of medicine (Spruit 2015). With the addition of a sound philological and anatomical background as well as new thoughts on method and how to apply it to the arts, Landi took the task a step further.

## Landi’s Paraphrase of the *De Anima*

**Fig. 2:**
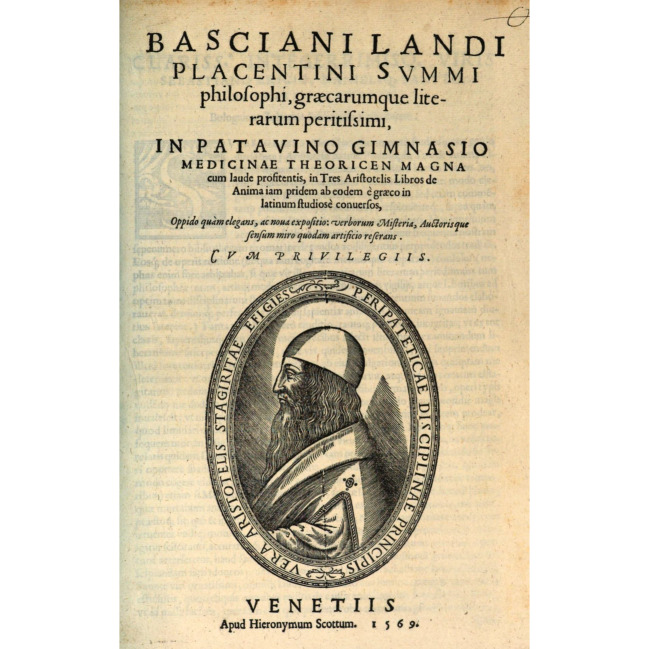
Frontispiece of Bassiano Landi’s commentary on Aristotle’s *De anima* (1569). Courtesy of the Bayerische Staatsbibliothek

Unlike Pomponazzi’s treatment of Aristotle’s *De anima*, Landi’s is set as a translation with a short paraphrase of the most difficult passages (Fig. 2). In this form, the work situates itself as more authoritative and objective than a commentary on the text—by its very nature, a genre open to debate and controversy. Separated into particles of text (*particulae*) first written in Greek then translated in Latin and briefly explained (*explanatio*), the book is a real “anatomy of Aristotle’s text” which matches the method we have already seen at work in Landi’s anatomical dictations. As to be expected in this context, Landi is persuaded that Aristotle himself set the precedent for the use of such a method; he offers an example of this at the beginning of Book II, where he is committed to reframing Aristotle’s definition of the soul as an instance of division. He begins the paraphrase of the first particle of Book II by stating that “division is the guide (*dux*) that leads to the definition to be discovered” (*divisionis inveniendae dux est divisio*). He then goes on to define the soul so that it fits his preconceived ideas about division as a method of discovery:

“The soul is the first perfection because is not an accident but is substance, and it is substance, not matter, because matter is brought to perfection and it is not a composite of matter and form, because the composite is brought to perfection by the form. But it is the form that brings to perfection. Perfection is of two kinds, first and second perfection ‘and the soul is not the second kind’ because it depends on the first. It pertains to the body, because the soul is not an incorporeal substance, but corporeal in that it is attached to the body. It pertains to the physical domain because it is not the form of an artefact, that is it brings to perfection not an artificial but a natural body. It pertains to an organic ‘body.’ ‘In fact,’ a natural body is either without organs, like stones and metals, or it has organs, like an animal body. But the soul is proper to an organic body endowed with life. An organic ‘body’ is either provided with life, like living beings and animals, or is devoid of it, like a corpse. The soul is the perfection of a body that is endowed with life.”^31^

One of the main problems with Landi’s commentary on this passage is that he does not seem to realise that Aristotle uses division not as a method of discovering the essence (τί ἐστι ψυχὴ) of the soul but as means of clarifying it in relation to his metaphysics, having already established, for each of these divisions, the preliminary conditions that allow the choice between alternative pairs. Indeed, in *De anima *Book I, Aristotle uses exactly the kind of dialectical reasoning that compares different definitions in order to cross out those which are either contradictory in and of themselves or do not match the phenomena of life as witnessed in direct observation. At the beginning of Book II, he therefore returns (πάλιν) to them from a different perspective to systematise the notions already established inductively.^32^ Therefore, Landi’s decision to rewrite Aristotle’s definition as an instance of division ultimately disregards the empirical underpinnings of Aristotle’s philosophy in favour of a more certain *a priori* approach. And yet, there is more.

In his explanation, Landi openly states that “the soul is corporeal” because it is attached to the body (*quoniam haeret corporis*), which does not follow the definition Aristotle provides for the soul as a life principle. As seen, in fact, while it is true that the soul perishes with the body because it is contingent upon the functions of the body, it is not correct to state that, for Aristotle, the soul is a corporeal substance. Aristotle defines it as “an original activity” (ἐντελέχεια πρώτη) and as “a form” (εἷδος), which means that the soul is a functional principle related to certain material conditions, not a material condition unto itself.^33^ As a functional principle, the proper ontological status of the soul is that “it is neither a body nor without a body.”^34^ An analogy that Aristotle uses to illustrate this point is that of an axe and its function. While the act of cutting something (say a tree) cannot be performed without the instrument, the action itself is not the same as the instrument.^35^Furthermore, functions cannot be regarded as material instruments as they preside over the formation of the organs and guide their developments teleologically although, depending on bodily instruments as to their implementation, they eventually decay and die with the body. The only exception Aristotle seems keen to admit is that of the intellect (ὁ νοῦς) which, capable of grasping the ultimate metaphysical structure of reality, can be separated from the body and survive the death of the individual^36^.

Landi’s definition of the soul as a corporeal substance instead reflects an open stance towards materialism and the mortality of the soul, which owes a debt to various interpretations of Aristotle definition of the soul, chiefly that provided by Alexander of Aphrodisia, who interpreted the soul as an organic principle of the body residing in the heart.^37^ According to Alexander, only one function of the soul is not shared with the body, and this is the intellect that Aristotle called “productive of knowledge” (ὁ νοῦς ὁ ποιητικός). By actually “touching” the simple notions and final causes of phenomena, this function of the soul is both eternal and immortal. At the same time, it is not really human either; according to Alexander, in fact, Aristotle is referring to the only entity that could exist *per se*, without a body and mental representations of any sort—that is: God.^38^

Alexander’s interpretation has been controversial since antiquity, in that, while it made sense of Aristotle’s overall metaphysical plan, it ended up denying the individuality of the cognitive process. It is nevertheless to this very interpretation that Landi openly and boldly makes recourse in his commentary to Book III. He contends that the cognition of the ultimate causes of phenomena is not inductive but deductive. As such, it presupposes an external cause that reveals the universal notion potentially contained in the individual instances grasped by the senses. This cause must itself be eternal, always thinking and always in actuality. However, humans cannot think without the use of mental images, and when they think of the ultimate cause of phenomena, they do so in a way that presupposes a certain activity by the senses. Thus, they cannot think of themselves as themselves in a proper way because human existence entails corporeality and thus contingency. The only entity that thinks of itself as itself, without the mediation of matter, is God, and thus the intellect that produces knowledge in us is God himself.^39^ For this reason, Landi continues, it is necessary that once the body perishes, our activity as thinking beings perishes too—because the organic instruments that set the preconditions for our knowledge no longer exist. As seen, this position entails a certain loss of individuality in the cognitive process, but it does so by identifying the human and the divine in a more direct way, all the while re-evaluating and providing a new meaning for human action in the world. If, on the one hand, humans are deprived of an afterlife, on the other hand, they are granted the possibility to establish certain conclusions that do not depend on religious dogma. The mediation between the sinful human and the omnipotent God of Christianity is severed. God himself is a finite entity, a conclusion that Landi had already reached in the *Opuscula*, aptly disguising it as a necessary implication of the Aristotelian system.^40^

Regardless of whether the hope in such a direct identification with God was illusory or not, it was a solution that scholasticism could not offer to scholars. It denied the concept of original sin—that is the ontological divide between creator and creature—and, with it, the need for the redemption which Christ gifted humanity by dying on the cross. Furthermore, it was considered a position belonging to gentiles, a community not initiated to the scriptures. In rejecting this stance, therefore, scholastic philosophers borrowed from Thomas Aquinas’ (1225–1274) *De unitate intellectus contra Averroistas*, wherein he pointed out that cognition, either individual or universal, is an individual process (*hic homo intelligit*) (Aquinas 1976: ch. 3, par. 62).^41^ As such, it is opened to contingency, not least because the world itself is contingent upon the will of God as to its existence. In the study of the arts, this stance legitimised the supremacy of Catholic theology over all faculties and disciplines, which received their subaltern status based on the proximity of their object of study to that of theology. Upholding the opposite, by advocating a direct identification of the human and the divine with the aid of a method that risked undermining the established curriculum, marginalising the scholastic rationale, and reclaiming more autonomy for the arts, was a very bold move indeed. In the midst of the Counter-Reformation, it was surely more than enough to attract academic malevolence and the charge of heresy.

This explains why Landi could not publish his lectures on the *De anima*, which circulated privately up to 1569 when they were finally printed and dedicated to his powerful Venetian pupils, Daniele Sanuto and Sebastiano Erizzo. A fate different from the one that befell Pietro Pomponazzi awaited Landi: this time, critics were ready to prevent the attempt at subverting Aristotle before it was too late, and they eventually succeeded. As to the authors of this attack, we cannot be sure, but it is easy to endorse the contemporary claim that it must have been those who had the most to gain from Landi’s death. In the light of this, we may attempt to draw a line between Landi and another heretical figure of the Paduan milieu—that one of Paolo Sarpi (1552–1623). Like Landi, but unlike Pomponazzi, Sarpi was interested in using philosophical authorities and a certain version of Aristotelian naturalism against the pervasive influence of the Catholic Church in matters of faith and politics. Yet, unlike Landi, Sarpi was not personally engaged in the academic arena. Both, nevertheless, seem to have attracted the same malignant forces, so that, to borrow from one of Sarpi’s most famous mottos, in the murder of the physician Bassiano Landi, contemporaries could indeed recognise “the style of the Roman curia.”^42^

## Footnotes

1 “Bassiano Lando Placentino, medico excellentissimo, theoricam medicinam in Gymansio Patavino profitenti, cum multi inviderent: inventi etiam sunt nebulones quidam qui vitae illius insidiarentur, anno Christi LXI. Urnulam pulvere incendiario replent, eamque sub cathedra, e qua singulis diebus publice declamare solebat, clam reponunt, funem longum, qui extra auditorium per occultas quasdam rimas protenderetur, urnulae adaptant, eo consilio, ut fune extra auditorium accenso, ignis sensim prorepens, pulverem in urnula contenum attingeret, et Bassianum cum summo auditorium suorum atque adeo extremo periculo collegae sui Junii Pauli Crassi, qui eadem hora in superioris contignationis aula profitebatur, e medio tolleret. Et sane voto suo potiebantur, nisi Bassianus non emunctae tantum naris homo, sed exquisiti quoque odoratus, ubi cathedram conscendit, fumosum quaendam halitum sensisset. Eum primo ex vicinis aedibus exurgentem susbicatur, cum nihil remitteret, famulo imperat, ut quid hoc rei esset, investigaret. Reperitur igitur funis accensus, cuius ductum secuti, urnulam sub cathedra sepositam vident, quae cum pulvere vitam simul et mortem Bassiani post sextantem horae clausisset. Venetias Bassianus proficiscitur, de insidiis conqueritur. Senatus illum bono animo esse iubet, et ut ad professionem revertatur, obsecrat. Indici duo millia librarum, et unius exulis revocandi potestas, decreta fuerunt. Nec tamen, quamvis amplissimis propositis praemis, auctor sciri potuit (Zwinger 1604: 1971).” This description parrallels the one provided by Gilbert Cousin (1567: 611–614). Another report of this event was discovered by Silvia Ferretto in a manuscript at the Ambrosiana Library in Milan, see Ferretto (2012: 19–20).

2 The cause of Landi’s death was first linked to an academic rivalry with scholastic philosophers by his disciple, Agostino Vailier (1531–1606) (Vailier 1719), and later by the Paduan physician Antonio Polo (Polo 1578). The same thesis was convincingly restated in Ongaro (1965: 265–278).

3 Reference to which can be found in Landi’s *Ekphrais de motu* in Landi (1552:13). See also Ongaro (1965: 266): “La fama di cui godeva e l’atteggiamento di superiorità e di disprezzo che ostentava, sia parlando che negli scritti, nei confronti di altri professori e specialmente di quelli *qui Latini, et scholastici nominantur*, gli suscitarono contro la malevolenza e l’odio, che armarono la mano del sicario.”

4 I would like to thank the author for allowing me to take a look at the proofs of his important work *Gabrielle Falloppia (1522/23-1562). The Life and Work of a Renaissance Anatomist*, and for sharing this alternative account of Landi’s death with me.

5 On Landi’s proximity to Pomponazzi’s interpretation see Polo (1578: 105): “Recordor, cum essem Patavii, quendam Gabrielem Albertum Pedemontanus, tribus mensibus quaestionem de immortalitate animae pertractasse, et in fine omnis resolutionis dixit ut ignarus, et impius, nisi esset Plato, tenerem mortalitatem animae. Similiter quidam *Bassianus Landus, qui privatim tertium Aristotelis de anima multis nobilibus scholaribus exponebat, ad mentem impii Pomponatii* dicebat, mihi displicet vobis legere impietatem, sed Aristotelis litera sic ait, et ipse sic tenet. Unde videns ego, et considerans has suas expositiones falsas esse, praesentibus omnibus scholaribus, per viam Epilogi, omnia sua argumenta reassumpsi et reieci. Et secundum veritatem Aristotelis exposui. Qui nolens respondere, sed tacens, dicebat, sic ait Aristoteles. Et deinde dedignatus, noluit amplius ut ego audirem suas falsas lectiones. Sed sicut falso veritatem docebant, *sic permittente Deo ambo ab incognitis trucidati fuere et sic ponas suae impietatis soluerunt*.” Italics added. For a fuller context of this quote, see Ferretto (2012: 21–23).

6 Landi (1552): Dedicatory letter to Francesco Contarini (17 March 1552).

7 Landi (1543): *Epistula nuncupatoria*, 3 [unnumbered]: “[N]ec philosophiae tantum inhædi, quin ad studia humano generi utiliora me avocari cognoscerem. In quibus, si aliquid sum futurus unquam, id omne IOANNI BAPTISTA MONTANO acceptum feram: tum quia is semper quam vis sponte currentem me excitavit, ut medicinam cum philosophia ad meam utilitatem coniungerem: tum etiam, quia propriam medendi rationem, quae tot ab hinc annis incognita fuit, iam biennium mihi cæterisque bonarum literarum studiosis commonstrat, atque sine fuco et fallaciis communicat.”

8 Landi (1543): *Epistula nuncupatoria*, 3 [unnumbered] and 18–20.

9 The first two letters (Erizzo to Landi, Venice, 15 November 1551 and 4 March 1552) were published in the sixteenth century by Atanagi (1571: 305v–309r), while a third (Erizzo to Landi, Venice 23 April 1556), dealing with Erizzo’s translation of Plato’s *Timaeus*, was recently rediscovered (Tomasi 2014). Erizzo’s epistulary has been edited and carefully contextualised by Claudia Marconato in Marconato (2018).

10 Sebastiano Erizzo to Bassiano Landi, Venice, 15 November 1551 in Atanagi ( 1571: 305v–307v).

11 Sebastiano Erizzo to Bassiano Landi, Venice, 4 March 1552 in Atanagi (1571: 308r–v).

12 A different consideration is offered by Ferretto (2012: 94–97), who pays little attention to the connection between Erizzo and Landi as to the question of the best method to teach the arts.

13 Erlangen-Nuremberg University Library, Ms 909, cc. 195r, 205r (n.d.).

14 Ibid., c. 160r.

15 Ibid., c. 166r.

16 See Vassaeus (1541). This is a very rare book; a copy can be found at Cambridge University Library, Classmark: N*.3.17(B)(6).

17 Ms 909, cc. 167r, 189r, 199v

18 Ibid., c. 171v.

19 Ibid., c. 181r: “haec omnia breviter nobis monstrabatur”; c. 182r: “et haec sunt quae de matrice breviter videre poteram.”

20 Ibid., c. 182r.

21 Ibid., cc. 187r, 189v–190r.

22 Ibid., cc. 168v, 172r, 186v.

23 Ibid., c. 175r.

24 “Videre licet Aristotelem, et eius interpretes, qui recentes sunt, accuratissime artem hanc examinasse, quam Anatome dicimus.”

25 “Illud autem dictis adijciendum est, ut in Commentariis de Anima declaratum est, dissimilitudinem in corpore omnem, quae naturalis est, ex magnitudine, et multitudine virium formae ipsius pendere.”

26 Aristotles, *De Anima* (= An.), 412a, 19–21: ἀναγκαῖον ἄρα τὴν ψυχὴν οὐσίαν εἶναι ὡς εἶδος σώματος φυσικοῦ δυνάμει ζωὴν ἔχοντος. ἡ δ’ οὐσία ἐντελέχεια’; see also 412a, 27–412b, 2: διὸ ἡ ψυχή ἐστιν ἐντελέχεια ἡ πρώτη σώματος φυσικοῦ δυνάμει ζωὴν ἔχοντος. τοιοῦτον δὲ ὃ ἂν ᾖ ὀργανικόν.

27 Ibid., An., 431a 14–17: τῇ δὲ διανοητικῇ ψυχῇ τὰ φαντάσματα οἷον αἰσθήματα ὑπάρχει, ὅταν δὲ ἀγαθὸν ἢ κακὸν φήσῃ ἢ ἀποφήσῃ, φεύγει ἢ διώκει· διὸ οὐδέποτε νοεῖ ἄνευ φαντάσματος ἡ ψυχή.

28 Aristotle, *Metaphysica* IX, 1051b, 17–22.

29 Aristotle, An. 402b, 20–25.

30 This occurs many times in Aristotle’s texts, as for instance An. 408b, 18–29. For the context and application of θέιος as related to νοῦς, see Burnyeat (2008).

31 Landi (1569): Particula IIII, Explanatio, p. 16v: “anima est perfectio prima, quia non est accidens, sed substantia, et substantia quidem, non materia, quia materia perficitur, nec compositum ex materia et forma, quia compositum perficitur a forma. Sed forma est quae perficit. Perfectio est duplex prima et secunda, non secunda, quia pendet a prima. Corporis, quia anima non est substantia incorporea, sed corporea, quoniam haeret corporis. Physici, quia non est forma artificiosi corporis, id est non perficit corpus artificiosum, sed naturale. Organici, corpus naturale aut caret organis, ut lapides, ut metalla, aut habet organa, ut corpus animalium. Sed anima est corporis organici vitam habentis. Organicum aut vitam habet, ut animantia et animalia; aut caret vita, ut cadaver. Anima est perfectio corporis vitam habentis.”

32 Aristotle, An. 412a, 3–5: Τὰ μὲν δὴ ὑπὸ τῶν πρότερον παραδεδομένα περὶ ψυχῆς εἰρήσθω· πάλιν δ’ ὥσπερ ἐξ ὑπαρχῆς ἐπανίωμεν, πειρώμενοι διορίσαι τί ἐστι ψυχὴ καὶ τίς ἂν εἴη κοινότατος λόγος αὐτῆς.

33 Ibid., An. 411b, 19–412b, 10.

34 Ibid., An. 414a, 19–20.

35 Ibid. An. 412b, 10–17.

36 An. 408b, 18–29: ὁ δὲ νοῦς ἔοικεν ἐγγίνεσθαι οὐσία τις οὖσα, καὶ οὐ φθείρεσθαι. μάλιστα γὰρ ἐφθείρετ’ ἂν ὑπὸ τῆς ἐν τῷ γήρᾳ ἀμαυρώσεως, νῦν δ’ ὥσπερ ἐπὶ τῶν αἰσθητηρίων συμβαίνει· εἰ γὰρ λάβοι ὁ πρεσβύτης ὄμμα τοιονδί, βλέποι ἂν ὥσπερ καὶ ὁ νέος. ὥστε τὸ γῆρας οὐ τῷ τὴν ψυχήν τι πεπονθέναι, ἀλλ’ ἐν ᾧ, καθάπερ ἐν μέθαις καὶ νόσοις. καὶ τὸ νοεῖν δὴ καὶ τὸ θεωρεῖν μαραίνεται ἄλλου τινὸς ἔσω φθειρομένου, αὐτὸ δὲ ἀπαθές ἐστιν. τὸ δὲ δια νοεῖσθαι καὶ φιλεῖν ἢ μισεῖν οὐκ ἔστιν ἐκείνου πάθη, ἀλλὰ τουδὶ τοῦ ἔχοντος ἐκεῖνο, ᾗ ἐκεῖνο ἔχει. διὸ καὶ τούτου φθειρομένου οὔτε μνημονεύει οὔτε φιλεῖ· οὐ γὰρ ἐκείνου ἦν, ἀλλὰ τοῦ κοινοῦ, ὃ ἀπόλωλεν· ὁ δὲ νοῦς ἴσως θειότερόν τι καὶ ἀπαθές ἐστιν.

37 Alexander of Aphrodisias, *De anima*, An. 10, 1–4 (the progression of organic forms) and 39, 19–23 (for the heart as the material seat of the entire soul).

38 Ibid., 89,17–21; 90, 13–20.

39 Landi (1569): Particula XV, Explanatio, 41r.

40 Landi (1552): *Ecphrasis de Motu*, 28v (unclear because of faulty pagination, it seems 36v)

41 Aquinas’ statement in context reads as follows: “Manifestum est enim quod hic homo singularis intelligit: nunquam enim de intellectu quaeremus, nisi intelligeremus; nec cum quaerimus de intellectu, de alio principio quaerimus, quam de eo quo nos intelligimus.”

42 The sentence is famous in Latin as *agnosco stilum Romanae Curiae*, wherein *stilum* can mean either style or stiletto and is attributed to Paolo Sarpi by his biographer Fulgenzio Micanzio (1646: 169): “dicendo l’Acquapendente non aver medicata ancora la più stravagante ferita, prontamente il Padre disse ‘E pure il mondo vuole che sia data *stilo Romanae curiae*’.”
